# Medical Education for Dentists

**Published:** 1888-12

**Authors:** C. M. Wright

**Affiliations:** Cincinnati, O.


### Medical Education for Dentists.

BY C. M. WRIGHT, D.D.S., M.D., CINCINNATI, O.

[Read before the Ohio State Dental Society, Cincinnati, October 16, 1888.]

I should be sorry to have this subject of the education of the dentist, or rather education for dentistry, become settled without having put my finger into the pie, metaphorically speaking. I have felt all along that there was no hurry about the matter. It will not be fixed until the "cohesive or non-cohesive gold" question, the "amalgam" question are fixed, and these of course we shall always have with us. But the education question like these others, might become quiescent for a while and I might not be here or not in condition to take an interest in it when it shall again appear on the tapis, so I seize this opportunity and plunge into the subject now.

The fact that busy and prominent practitioners of medicine and dentistry, who are not often professors in medical or dental colleges, or editors of medical or dental periodicals, are so much interested in the education, or proper preparation of those who are destined to occupy the shoes of the present race of practitioners, must have some reason. There must be some grounds for the discontent with past and present methods of education. What are these grounds? Why are Drs. A, B, and C, who have been successful in gaining professional favor, professional honors, and large practices, where one would suppose all their energies could be profitably engaged, not contented to let the coming doctor and dentist work out his own salvation, in the same way that they themselves have? It cannot be that Drs. A, B, and C, are possessed by the foolish idea, that "now that we are in, we must make it as difficult as possible for others to get in." It can not be that Drs. A, B, and C, who have done so nobly in all other respects, can have any but the purest motives in this direction. They are doing what they consider a duty, and this duty lies at present in trying to make the fence about the dental field, higher and higher, and in some cases adding a barbed wire at the top of the fence. Why this should have become a duty, I cannot answer. I must acknowledge that I do not know; nor can I see any reason or sense in it. This may be my fault, but I am open to conviction. Fences are good in their way for keeping out intruders and for defining boundaries. When the distinguished Drs. A. B. and C entered the profession, the fences were so low and so badly built that they could be stepped over with the greatest ease, and no thoughts of the danger of rents and tears from the abominable barbed wire ever entered the intruder's mind.

Gentlemen, to be sincere, the fence was a very low one when I entered the profession. The requirements were a two year's apprenticeship or studentship, and a course in a dental college. The labor, the anxiety, the sleepless nights and worried days, the study of medical science, all came afterwards when securely within the field. For several years the mechanics of our practice occupied my best thoughts. I don't think I looked or thought of anything beyond the *how* to do it, in daily practice. The *how* is just as important to-day as it was a quarter of a century ago. Clinical experience seemed to me the very first thing of value, and to acquire that required all the energy and devotion possible. It is to-day as valuable and as difficult to acquire as at any past time in our history. Cultivation of the muscles so that they shall respond delicately and skillfully and accurately to efferent impulses from a trained mind is of the first importance in our specialty. Will a medical education train the mind for this sort of work? If I should answer the question in my own way, I should say, "not particularly." Latin, Greek, the higher mathematics, the natural sciences, all furnish gymnastic exercises for general mental development. So does the study of the science of medicine. The particular study which the dental student needs is exactly that which the best class of dental colleges of to-day offer with their two courses of lectures on anatomy, chemistry, materia medica, physiology, pathology, general and special, and therapeutics, and their present facilities for and encouragement of diligent laboratory and infirmary practice. This is all that the beginner needs. Nothing more, nothing less. It is the education *par excellence*, for the preparation of young men who propose to enter the field of dental practice. It is practical. It is the outgrowth of independent American directness of purpose, untrammeled by ancient precedents. It has been evolved from American necessity, and has been admired and applauded and copied (as near as possible ) by special educators in other countries. We hug ourselves on account of the reputation our young specialty has gained in the world. The dental college is the prime factor in the good reputation. The dental college standing alone and independent of any medical faculty. The American Dental College, which has become an institution, in the broader meaning of that term, in our country and only in our country. This is the school for the dental student. It isn't the school for medical, or law, or theological students. Neither are medical, or law, or theological schools the place for dental students.

But the science of dentistry we say, is a branch of the science of medicine. Medicine is the *alma mater* of dentistry as well as ophthalmology, gynæcology, etc., etc. This is all true, and I for one have no disposition to dispute the proposition in any of its points, but when you go on to say after stating the above proposition, " therefore, the dentist like the oculist should study medicine first and dentistry afterwards," I object positively, and insist that the conclusion has no logical connection with the accepted proposition. Anatomy is a branch of the science of biology. Would it not be better for a young man who expects to become a practical anatomist to begin with dissections and the study of anatomy proper, than for him to spend a year or two in the study of biology? After the technique has been acquired and skill with the scalpel, how natural it is for the student of anatomy to extend his studies and reach back into the interesting science of biology. I don't want to multiply examples, but to insist that the study of medicine becomes a desire, a pleasure, a necessity to the dentist *after* the course in the dental college, and after manual skill has been attained in the infirmary and in practice. Drs. A, B, and C have pursued this course. Dental graduates are continually supplementing their dental studies by later medical studies. A dozen dental graduates in the immediate vicinity of this house have within a few years graduated at one or another of the medical colleges of this city. It is not at all an uncommon occurrence, and what fine medical students the dental graduates make! Medical education for *dentists* is of the greatest advantage. Medical education for dentists is becoming every day a greater necessity. We must all approve of it. But I want to make the distinction as clear as the line between black and white, that medical education for *dental students* is as unnecessary and as unprofitable, as the study of theology would be to a law student. It would be a waste of physiological energy at the wrong time. It is not a direct route. It is dilletantism and harmful, instead of useful to practical students. A knowledge of the use and skill in the use of the forceps, or laryngoscope, and of any of the special instruments of specialists in surgical practice, can only be acquired by a special training, whether after a medical course or before a medical course.

The dentist, among all specialist, is the only one who has a special school devoted to this necessary training. The school has proved a success. Why then should we want to change its character and go back to older and inferior methods? Ophthalmology and laryngology might be very much benefited if these specialties had their schools arranged exactly after the pattern of the dental colleges, and entirely independent of medical colleges. This may be but a matter of opinion. Many things are but matters of opinion.

"By their fruits ye shall know them." The fruit of the American Dental College has made American dentistry preeminent in the world. The medical colleges have ripened but very little fruit in the dental orchard. If you will show me one very prominent and distinguished practitioner of dentistry who has come to us from the medical schools, I will show you ten equally as prominent and distinguished who have come from the dental schools. Dr. Norman Kingsley looked into this subject some years ago, and stated some significant facts in regard to it. We might adopt his plan and analyze this assembly as a representative society of the State of Ohio. How many of us members of this society have received a medical college education first and our dental education afterwards? I suspect a straw vote would show a very insignificant minority of the former. Why then, gentlemen, do Drs. A, B, and C want to change the methods of the dental college, or attach dental *chairs* to medical college faculties? These dental *chairs*, so-called, have always seemed to me to bear about the same relation to a chair in an independent dental school, that a little wicker work child's chair with a hole in the bottom, does to a comfortable adult arm chair. Our profession is too old for the little chair now. Let's put it away in the garret with the tin soldiers, rag-babies, and rocking-horses of our babyhood, and stick manfully to our dental schools and the chairs filled by professors of the science and art of dentistry.

DISCUSSION OF DR. WRIGHT'S PAPER.

*Dr. Berry*—Dental colleges were commenced when our country was new. The Baltimore College was founded about half a century ago. Not long since at a State dental society a dentist, who did not know what he was talking about, said that the four professors of this college spent the entire time of the first session in teaching mechanical dentistry only; while, in fact, eminent in their professions for their talent and learning, gave probably as thorough and able a course of lectures on the subjects of their chairs as has ever been given in any college since.

Five years after the founding of the Baltimore College, and only about fifty years after the Indian was driven out of this valley, the Ohio College of Dental Surgery was established. The teaching of the first term was very good; and, although there was no professorship of chemistry, Dr. Slack, one of the charter members of the Medical College of Ohio, gave, on this subject, an able course of lectures.

As time passed, improvements were made in the course of instruction in the dental college, and they are doing good work for the profession. But too short time of study and attendance at college is required. There is too much for the average student to learn, for him to master it, in two of the present college terms. Of course, as this need of more thorough acquirement is felt by our profession generally, the standard of attainment for graduation will be raised.

How many chemists are there in the dental and medical professions in Ohio? Are there twenty? I do not mean those who can tell the number of the elements, or describe oxygen, but who are well posted in chemical science. The dentist or physician who is not proficient in chemistry is very lame.

*Dr. Butler*—I am not at all surprised that the gentlemen connected with dental schools directly, should be a little concerned in talking up this subject; and we would all be glad to hear from them.

The paper of Dr. Wright reminds me of one presented by Dr. Tomes, at the International Medical Congress, held in London, in 1881; he placed himself in the same position that Professor Tomes did, in regard to medical dentistry; he spoke for the students and I agree with him. There are those here, too, who know that I have been in favor of a dentist being also a graduate in medicine, and I hold that that position is perfectly right as stated; it is a valuable acquirement. Professor Tomes said that education for dentistry was similar to the training of the young man that wanted to acquire facility as a musician in the handling or the manipulating of a musical instrument. If he ever becomes an expert on the piano, organ, or violin, or on any of these instruments, he must do it in his younger days while he has full use of his digital powers. Now in order to become a skillful dentist a young man must educate his hands and fingers. I once read a paper before this society on the education of the head and hands in order to become a dentist. Now why has dentistry advanced so rapidly in its short life as a profession?

In all medical schools where they have the highest status they provide for that. They have the greatest of clinical facilities in the hospital, in the amphitheater, where the student can have his didactics brought down in the practice, and here is where the dental schools are pre-eminent in their mode of teaching, and those that have the best hospital or infirmary department, or clinical department, where all these different manipulations may be gone through under the directions of an able teacher in that department. These are the best preparations for those who intend to practice dentistry, and the school that fails to provide for that thing in the very best possible manner is not doing what it ought to do, and the medical school that is turning out graduates year after year with very poor facilities in that direction fails to give us the best recruits for the profession.

You take any of these specialists and if they become expert manipulators you will find that they commenced their study in that particular direction in their younger days. You may look the world over in any department, and you will find that these men are those that commenced the study of that particular field in their younger days. As Dr. Wright has said, after they have acquired a dental education they have to study medicine in the different departments. This is all valuable, very valuable, they are indispensable in fact, and whether his Mr. A., B. and C. acquired their knowledge easily or through a hard field makes no difference. If you want to become a practitioner for your own honor and the honor of the profession, and to obtain a prominent place among them you must expect that you have hard work before you, and you will not succeed unless you are energetic and set yourself to work with a determination that no man shall exceed you in your calling.

*Dr. Fletcher*—I was thinking of defending this chair spoken of in Dr. Wright's paper. I think he has put that chair in a very bad light. You probably have all had occasion to observe that dentistry has attained that position in which we might very easily give instructions on diseases of the teeth and mouth to many a professor in medicine. Those of you who have had any experience in that line know how many times patients having a diseased tooth, may be prostrated for days being attended by physicians who have little knowledge of how to relieve them from such pain. And for that reason if no other, I would hold that a dental chair in a medical college would be a great benefit to both physician and patient, at least the physician should know when to send a patient to the dentist, when proper occasion requires.

I look upon the profession of dentistry as requiring some medical knowledge, and I think that students ought to have a broader education than they simply receive in any one college.

In order to be successful in any line it is necessary at least to have some knowledge in many other branches, and the more knowledge you have on any branch which bears directly or indirectly on the line of investigation which you are following, the better you are fitted for the work. By a surplus of knowledge we certainly do our work more easily, with greater benefit to our patients than by working up to our full capacity in very ordinary operations. I for one should be glad to see—if it could be arranged in a practical way—the standard of education elevated in the dental profession. I have no complaints to make of the dental colleges and their standing, but I do hold that a man can simply graduate at a dental college and not be fully competent to attend intelligently to every case which comes into his office. I look upon a dentist who has some knowledge of medicine as more competent to practice than without such knowledge, but I would not reverse the order of things. My idea of the education for the dentist would be that you should start out with the practice of dentistry, and not that you should start out to practice medicine, and then if you make a failure of that to try dentistry afterward. I would like to see the standard of our profession so elevated, not that we should keep out those who would like to come in, but they should fully realize what it requires to be a successful practitioner. What I mean by a successful practitioner, is not in a financial point of view, but that we be so educated that when a case is brought before us we may fully comprehend what the case requires, and I should say that in order to do this it is necessary that we should have some outside knowledge besides that we have received at any one college or from any one line of study.

*Dr. Taft.*—In reference to the paper, the author seems to take it for granted that there is a general or at least a commonly entertained opinion, that it is important that a man seeking to enter the practice of dentistry should first graduate from a college of medicine. I do not think that such an opinion is general. I know that a few persons have entertained that opinion, but I know also that others have indicated some modifications within a reasonable period, therefore, it seems to me that it was hardly necessary that the paper should present that point so prominently, that there were people or members of the profession, and others perhaps entertaining the idea that every man who seeks to enter the dental profession ought first to be a graduate of medicine.

Now in regard to the amount of knowledge that ought to be possessed by one practicing dentistry there perhaps would not be a great difference of opinion. Among intelligent men in a profession I think there would be a much nearer agreement of opinion than many suppose. How they should get that knowledge perhaps is a question of considerable importance, but this may be borne in mind that the capabilities of different individuals are not alike, but of course in a matter of this kind the proper course to pursue is that which best serves the largest number. Now I do not believe that one starting into preparation for the practice of dentistry, a young man of eighteen or twenty years of age, ought to take up a regular course of medical instruction as fully as one who intends to make the practice of medicine his life work. It seems to me that the better method is to train the manipulative faculties and that these should first have attention. During the period of life from eighteen to twenty-four, or perhaps earlier, the manipulative faculty will be more easily cultivated than at a later period of life. I am very fully impressed that this training should begin early and should be taken up at the beginning of the work. The teaching of principles should in one sense follow the technical training. Some students are able to carry forward a number of branches with profit, while others will struggle and labor hard with two or three and some with one. The proper course is for such a person to be guided according to that which he is able to do, and let him accomplish that thoroughly as he proceeds. The graded system of study and work should be adopted by all colleges.

As to graduating first in medicine I do not believe it the better course. Whether a man graduates in medicine after he has pursued a dental course rests with himself, and if he possess the knowledge it is not a matter of very great importance whether he has received the degree or not. A degree is simply a certificate of those who have been the teachers, and it only means that the person receiving the certificate has pursued a certain course of study up to a certain point.

Now, as to teaching dentistry in medical colleges—this was referred to—I do not understand that this is advocated by anybody. I do not know of any medical colleges that have dental chairs with a view of preparing students to practice dentistry; it is possible that there may be some but I do not know of any. The foundation branches of medicine should be studied by every dentist, but as to the best methods of doing this there may be differences of opinion. Of course every dental college should be organized so as to embrace all of these branches.

*Dr. Smith*—I am very happy to learn by Prof. Taft that there is not a great difference in regard to the method of educating dentists. I thought there was a wide difference. Of course I am a little modest in regard to expressing my views. I recall an address made by Prof. Hopkins, who was a very eminent educator, a man of education as a lecturer. He said that it was a waste of time to burden the education of the specialist at the time that he was fitting himself, and he said that he would rather know every thing about some thing than some thing about every thing. So it is with dentistry. You may know every thing about dentistry; make that a specialty in the beginning, and then if one has acquired that little, let him complete his education. Now I deny most emphatically that the dental colleges of to-day do not educate a man thoroughly. The public do not expect us to treat those diseases which have been referred to, with the diseases of the teeth. We refer those cases to the medical men; we recognize the disturbances, but when we pass that we are beyond our domain as dentists, of the recognized domain ot the practitioner of to-day. The dentist ought to have a knowledge of biology and geology; he must have a knowledge of anatomy, and these branches are taught in dental schools; and it is taught just as thoroughly, in my estimation, as it is in the better class of medical colleges.

*Dr. Gray*—I wish to speak from a little experience in regard to the paper just read by Dr. Wright. It pleased me very much, as far as I am able to judge. I like the sentiment expressed in the paper. Physical training will help the muscle of the individual, and also that the mental training would benefit the individual in any pursuit which he may follow. The old adage has it, that education will aid any one in any pursuit or any profession he may wish to follow. A great many young men when they graduate in dentistry, do not have the means to graduate in medicine. I know I had not, and so it is with a great many others. But I must say it would be a good thing, but we must do the best we can. A young practitioner, who has just graduated, generally is not rushed with business; and while I was sitting in my office after my graduation, I gave all my spare time to the study of medicine in order to enlighten myself in the profession. A young man has plenty of time to study the medical branches in his office during his leisure hours after he has gone through with the dental course. I may say from my experience in dentistry that we do not need a great deal of medical science. When I first started out in life I thought I would study the medical profession, and before attending lectures I read medicine a little and I found that to get to the top of the ladder I would have to be a life-time student, and I have found the same thing in dentistry.

*Dr. Harrison*—I am very much in favor of having a high aim in all callings of life, and while that is the case with the dental colleges that we have in the United States for the education of dentists, and no better advantages in a medical college for the education of physicians, I do not think it is the wise plan to hold out before the students that it is necessary to go to a medical college after they get through with the dental college. I believe that you get all in a dental college that is necessary in the practice of dentistry. Let us not feel that we as dentists are below the medical profession. When I want to practice dentistry, I practice dentistry, and when I want to practice medicine I practice medicine.

